# Histologic Inflammation can Predict Future Clinical Relapse in Ulcerative Colitis Patients in Endoscopic Remission

**DOI:** 10.1093/crocol/otad059

**Published:** 2023-10-18

**Authors:** Lauren A George, Harris T Feldman, Madeline Alizadeh, Ameer Abutaleb, Samantha Zullow, Ashley Hine, Kristen Stashek, Suparna Sarkar, Katherine Sun, David Hudesman, Jordan Axelrad, Raymond K Cross

**Affiliations:** Division of Gastroenterology and Hepatology, Department of Medicine, University of Maryland School of Medicine, Baltimore, MD, USA; Department of Medicine, Department of Pediatrics, University of Massachusetts Medical School, Worcester, MA, USA; Division of Gastroenterology and Hepatology, Department of Medicine, University of Maryland School of Medicine, Baltimore, MD, USA; Institute for Genome Sciences, University of Maryland School of Medicine, Baltimore, MD, USA; Division of Gastroenterology and Hepatology, Department of Medicine, University of Maryland School of Medicine, Baltimore, MD, USA; Division of Gastroenterology and Hepatology, Inflammatory Bowel Disease Center, Beth Israel Deaconess Medical Center, Harvard Medical School, Boston, MA, USA; University of Connecticut School of Medicine, Farmington, CT, USA; Department of Pathology, University of Maryland School of Medicine, Baltimore, MD, USA; Department of Pathology, NYU Langone Health, NYU Grossman School of Medicine, New York, NY, USA; Department of Pathology, NYU Langone Health, NYU Grossman School of Medicine, New York, NY, USA; Division of Gastroenterology, Department of Medicine, Inflammatory Bowel Disease Center at NYU Langone Health, NYU Grossman School of Medicine, New York, NY, USA; Division of Gastroenterology, Department of Medicine, Inflammatory Bowel Disease Center at NYU Langone Health, NYU Grossman School of Medicine, New York, NY, USA; Division of Gastroenterology and Hepatology, Department of Medicine, University of Maryland School of Medicine, Baltimore, MD, USA

**Keywords:** ulcerative colitis, histology, relapse, remission

## Abstract

**Background:**

In ulcerative colitis (UC), endoscopic improvement, defined as a Mayo Endoscopic Score (MES) of 0 or 1, is a target of treatment. The aim of our study was to evaluate the risk of clinical relapse between patients with an MES of 0 or 1 and determine if histologic activity using the Robarts Histopathologic Index (RHI) was predictive of clinical relapse.

**Methods:**

UC patients with an MES score of 0 or 1, no prior colectomy, and at least 1 year of outpatient follow-up after colonoscopy were included. Demographic, clinical characteristics, and clinical relapse were retrospectively collected. Biopsy specimens were read by a gastrointestinal pathologist. Primary outcome was defined as a composite of relapse requiring change in medical therapy, new steroid use, UC-related hospitalization, and/or colectomy.

**Results:**

Four hundred and forty-five UC patients were identified. Ninety-five percent of patients with MES 0 were in histologic remission by the RHI whereas only 35% of patients with MES 1 were in histologic remission. Twenty-six percent of patients experienced a clinical relapse; patients with MES 1 or RHI > 3 were significantly more likely to relapse (*P* < .01) compared to patients with MES 0 or RHI ≤ 3. When patients were stratified into 4 groups (MES 0, RHI ≤ 3; MES 0, RHI > 3; MES 1, RHI ≤ 3; MES 1, RHI > 3) and adjusted for age and sex, RHI > 3 was predictive of relapse (*P* = .008).

**Conclusions:**

UC patients with endoscopic improvement have a high rate of clinical relapse over time. Histologic activity is a predictor of clinical relapse.

## Graphical Abstract

**Graphical Abstract:**
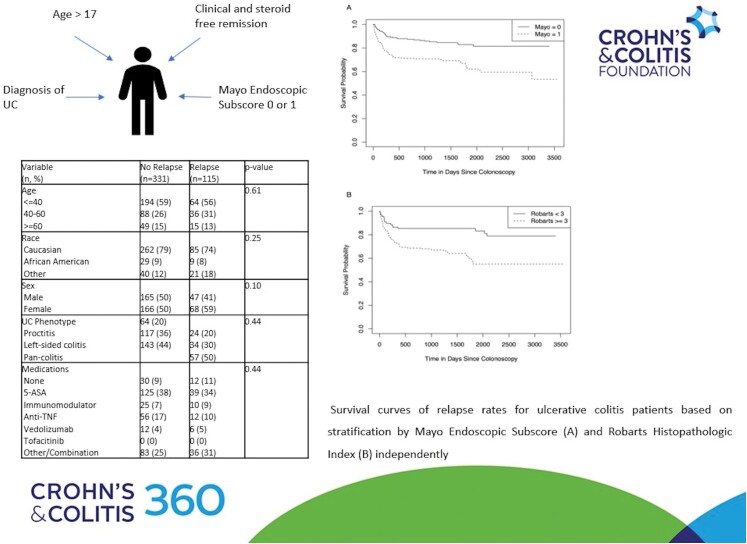

**Key Messages**

What Is Already Known: In ulcerative colitis (UC), a Mayo Endoscopic Subscore of 0 or 1 is considered endoscopic remission and is associated with a decreased risk of future clinical relapse.
What Is New Here: Despite endoscopic remission, the presence of histologic inflammation as defined by the Robarts Histopathologic Index, predicts an increased risk of future clinical relapse.
How can this study help patient care? Physicians can use histologic indices to inform the prognosis of UC patients in endoscopic remission, and stratify the need for enhanced monitoring protocols.

## Introduction

Inflammatory bowel disease (IBD) is a chronic inflammatory condition of the gastrointestinal tract characterized by periods of active disease and remission. The 2 major subtypes of IBD are Crohn’s disease (CD) and Ulcerative Colitis (UC), both of which can present with symptoms of abdominal pain, weight loss, and diarrhea.^1^ The relapsing-remitting disease course of IBD results in flaring of symptoms, often requiring escalation of medical treatment, hospitalization, and potentially surgical intervention. The prevalence of IBD in the United States is 3.1 million people, with 3 times more patients diagnosed with UC than CD.^2^ The management of IBD is complex, often requiring endoscopic assessment to monitor disease progression. Results from endoscopic examinations give the gastroenterologist endoscopic and histologic data, both of which can be used to assess for active inflammation.

Currently, endoscopic remission in UC is defined using the Mayo Endoscopic Subscore (MES), with a score of 0 (normal mucosa) and endoscopic improvement is defined as an MES of 0 or 1 (erythema, decreased vascular pattern) representing mucosal healing.^3^ Many studies have evaluated clinical outcomes in patients with quiescent versus active colitis using various endoscopic indices^4–6^; however, there is limited data comparing relapse rates of patients in endoscopic remission based on an MES of 0 versus 1. Though both scores would be considered endoscopic improvement, a 2016 small retrospective study of 138 patients diagnosed with UC found a significantly increased risk of relapse in patients with MES 1 compared to 0,^7^ indicating that patients with mild endoscopic activity have an increased risk of relapse over those that have no endoscopic activity.

In this study, we aimed to determine if UC patients in endoscopic improvement with an MES of 1 have an increased risk of subsequent UC relapse compared to patients with an MES of 0, considered to be in remission. There is currently a lack of data stratifying risk of disease relapse in patients who are in endoscopic remission. As such, we additionally aim to determine if UC patients in endoscopic remission with active histologic disease by the Robarts Histopathology Index (RHI) have an increased risk of UC relapse compared to patients in histologic remission.

## Materials and Methods

### Patient Selection

Our study was a retrospective chart review from the IBD centers at University of Maryland School of Medicine and New York University Langone Health, tertiary academic centers comprising a geographic and racially diverse population. Patients were identified through a query of the electronic medical record (EPIC) and endoscopy reports were reviewed. Eligible patients for this study had a confirmed diagnosis of UC based on standard clinical, endoscopic, and pathologic criteria and who underwent colonoscopy between January 1, 2012, and August 10, 2017. MES for the first colonoscopy in the electronic medical record must have been 0 or 1 for each patient to be eligible. If a MES was not provided in the report, descriptive findings of the endoscopist and de-identified images from the procedure were reviewed by an IBD-specialized gastroenterologist, and a MES was assigned to the images. Other inclusion criteria included no prior colectomy and at least 1 year of outpatient follow-up after colonoscopy. Exclusion criteria included patients with MES of 2 or 3, any IBD diagnosis other than UC, less than 1 year of follow-up after colonoscopy, corticosteroids within 30 days of colonoscopy, and colectomy prior to colonoscopy.

### Data Collection

Demographics, disease extent, smoking status, medications, inflammatory markers, and outcomes including clinical relapse were collected. Colonoscopy date and MES were documented, and patient outcomes were queried from the date of colonoscopy through May 1, 2020. Biopsy specimens from each colonoscopy collected for routine clinical care were read by expert IBD pathologists (KrS, SS, and KaS) to determine the RHI. As a retrospective study, biopsies were not protocolized and were collected at the discretion of the endoscopist; however, in both centers, it is standard practice to biopsy the areas of most severe inflammation. The highest RHI from each patient biopsy set, correlating with more severe inflammation, was used in final data analysis. Histologic remission was defined as an RHI of 3 or less.^8,9^ The primary outcome was defined as a composite of relapse requiring change in medical therapy, new steroid use, UC-related hospitalization, and/or colectomy within one year from index colonoscopy. Relapse requiring change in medical therapy was defined as any increase in UC symptoms requiring change in therapy such as from aminosalicylates to biologics. New steroid use was defined as a course of steroids secondary to an increase in UC symptoms. UC-related hospitalizations were defined as an increase in symptoms requiring any visit to emergency department with subsequent admission to the hospital. Colectomy was defined as surgery for patients with medically refractory disease and did include those that required colectomy for UC-related malignancy. The above clinical outcomes were subsequently evaluated through all follow-ups available in the medical record until May 1, 2020.

### Statistical Analysis

To determine which variables were associated with the composite outcome, baseline demographic and disease-related data for all patients were compared. The outcomes (IBD hospitalization, colectomy, steroid use, or relapse requiring change in medication) were combined in a composite fashion as a categorical variable. The composite outcome was assessed between groups using the Fisher’s exact and Pearson’s chi-square test. Potential covariates that may predict these outcomes were compared. Assessment for confounding was done by separately adjusting for co-variates in a logistic regression model. Clinical outcomes for patients stratified strictly by MES or RHI were assessed in an extended follow-up through Cox proportional hazards models, and logistic regression, controlling for time in study, was performed to assess differences in relapse likelihood across groups stratified by both MES and RHI (MES 0, RHI ≤ 3; MES 0, RHI > 3; MES 1, RHI ≤ 3; MES 1, RHI > 3). Cox proportional hazards models were not used to assess these differences across those 4 groups due to violation of the proportional hazards assumption, seen in the crossing of curves in Supplementary Figure 1, which was not seen when each (strictly RHI vs MES) was assessed independently (Figure 3). The logistic regression used for All modeling for extended clinical outcomes were performed in R version 4.0.4 using the “stats” and “survival” packages.^10,11^

## Results

### Demographics

In combined analysis from both IBD centers, 445 UC patients were identified. Of these, 228 patients had an MES of 0 (51%) while the remaining 218 (49%) had an MES of 1 on the index colonoscopy. Seventy-eight percent were Caucasian, 52% were female, and mean age was 40 +/−15 years at the index colonoscopy. Twenty percent, thirty-four percent, and forty-six perecnt had proctitis, left-sided colitis, and extensive or pan-colitis, respectively. Forty-six percent of patients were on either an aminosalicylate alone or no medical therapy, 46% were on biologics, and 27% were on biologic therapy with an immunomodulator (Table 1). Of all patients, 115 (26%) subsequently relapsed over 1 year from the index colonoscopy. Further demographics stratified by subsequent UC disease relapse can be found in Supplementary Table 1.

**Table 1.** Demographic and clinical characteristics of patients with ulcerative colitis at the University of Maryland and New York University Langone Health at time of baseline colonoscopy stratified by subsequent disease relapse.

Variable (*n*, %)	No Relapse (*n* = 331)	Relapse (*n* = 115)	*P*-value
Age			.61
≤40	194 (59)	64 (56)	
40–60	88 (26)	36 (31)	
≥60	49 (15)	15 (13)	
Race			.25
Caucasian	262 (79)	85 (74)	
African American	29 (9)	9 (8)	
Other	40 (12)	21 (18)	
Sex			.10
Male	165 (50)	47 (41)	
Female	166 (50)	68 (59)	
UC Phenotype			.44
Proctitis	64 (20)	24 (20)	
Left-sided colitis	117 (36)	34 (30)	
Pan-colitis	143 (44)	57 (50)	
Smoking			.68
Never smoker	230 (70)	80 (70)	
Former smoker	83 (25)	31 (27)	
Current smoker	18 (5)	4 (3)	
Medications			.44
None	30 (9)	12 (11)	
5-ASA	125 (38)	39 (34)	
Immunomodulator	25 (7)	10 (9)	
Anti-TNF	56 (17)	12 (10)	
Vedolizumab	12 (4)	6 (5)	
Tofacitinib	0 (0)	0 (0)	
Other/Combination	83 (25)	36 (31)	
ESR (mm/hr)			.11
≤30	31 (82)	12 (100)	
>30	7 (18)	0 (0)	
CRP (mg/dL)			.12
≤3	136 (86)	38 (76)	
>3	22 (14)	12 (24)	
UC duration (years)			.08
<10	178 (58)	76 (68)	
≥10	127 (42)	36 (32)	

### Baseline Activity

**Figure 1.**
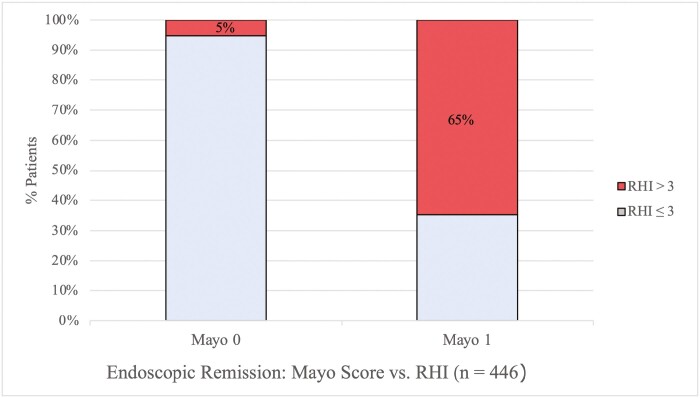
Robarts Histopathologic Score from biopsies taken in patients with ulcerative colitis at the University of Maryland School of Medicine and New York University Langone Health in endoscopic remission with Mayo Endoscopic Score 0 (*n* = 228) or 1 (*n* = 218).

At the time of their index colonoscopy, 228 had an MES of 0 at the index colonoscopy while the remaining 218 had an MES of 1. Histologic remission, defined by RHI as less than or equal to 3, was seen in 215 (95%) of 227 patients who scored as MES 0 at the index colonoscopy and had histology available. Within the MES 1 group, 77 (35%) of 218 patients had an RHI less than or equal to 3 (*P* < .01). (Figure 1).

### Clinical Outcomes at 1 Year

**Figure 2.**
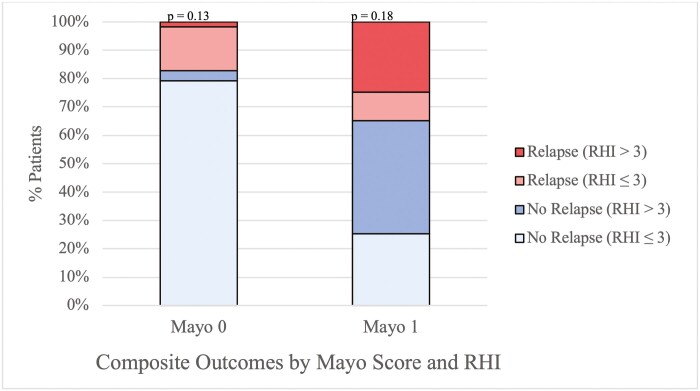
Clinical relapse at 1-year stratified by Robarts Histopathologic Index for patients with ulcerative colitis at the University of Maryland School of Medicine and New York University Langone Health in endoscopic remission with Mayo Endoscopic Score 0 (*n* = 228, *P* = 0.13) and 1 (*n* = 218, *P* = 0.18).

Within 1 year from index colonoscopy, 115 (26%) patients experienced the composite outcome of clinical relapse as defined by at least one of the following: Change in medical therapy, corticosteroid prescription, hospitalization, or colectomy. Of the 115 patients, 97 (84%) had a change in their medical therapy, 66 (57%) were prescribed corticosteroids, 8 (7%) underwent a hospitalization related to UC, and 4 (3.4%) patients underwent colectomy. A significant increase in composite outcome was found in patients with an MES of 1 versus 0 (Table 2). Of the 39 patients with an MES 0 that experienced relapse, 10% (*n* = 4) had an RHI greater than 3 at index colonoscopy while the remaining 90% (*n* = 35) had an RHI of less than or equal to 3 (*P* = .13). Of the 76 patients with an MES of 1 who relapsed within 1 year, 71% (*n* = 54) had an RHI greater than 3 (*P* = .18) (Figure 2). Regardless of MES, patients with RHI greater than 3 achieved the composite outcome of clinical relapse more frequently than those with RHI less than or equal to 3 (*n* = 58/153 vs. *n* = 16/88, *P* < .01). On initial univariate analysis, female gender and duration of disease trended towards significance. When adjusting for endoscopic activity by MES, female gender, and UC duration, an RHI of greater than three was associated with a trend towards increased risk of relapse although the confidence interval crossed 1 (CI 1.99, 0.98–4.05) (Supplementary Table 2). There was no difference in relapse risk based on the type of medical therapy at time of colonoscopy (non-biologic therapy vs. biologic therapy) or based on anatomical extent of disease.

**Table 2.** Risk of clinical relapse by Mayo Endoscopic Score and Robarts Histologic Index in patients with ulcerative colitis at the University of Maryland School of Medicine and New York University Langone Health by MES and RHI at 1 year.

Variable (*n*, %)	No Relapse (*n* = 331)	Relapse (*n* = 115)	*P*-value
Mayo endoscopic score			<.01
0	188 (57)	39 (34)	
1	142 (43)	76 (66)	
Mayo 0 score			.13
With Robarts > 3	8 (4)	4 (10)	
With Robarts ≤3	180 (96)	35 (90)	
Mayo 1 score			.18
With Robarts > 3	87 (61)	54 (71)	
With Robarts ≤3	55 (39)	22 (29)	

### Extended Clinical Outcomes

**Figure 3.**
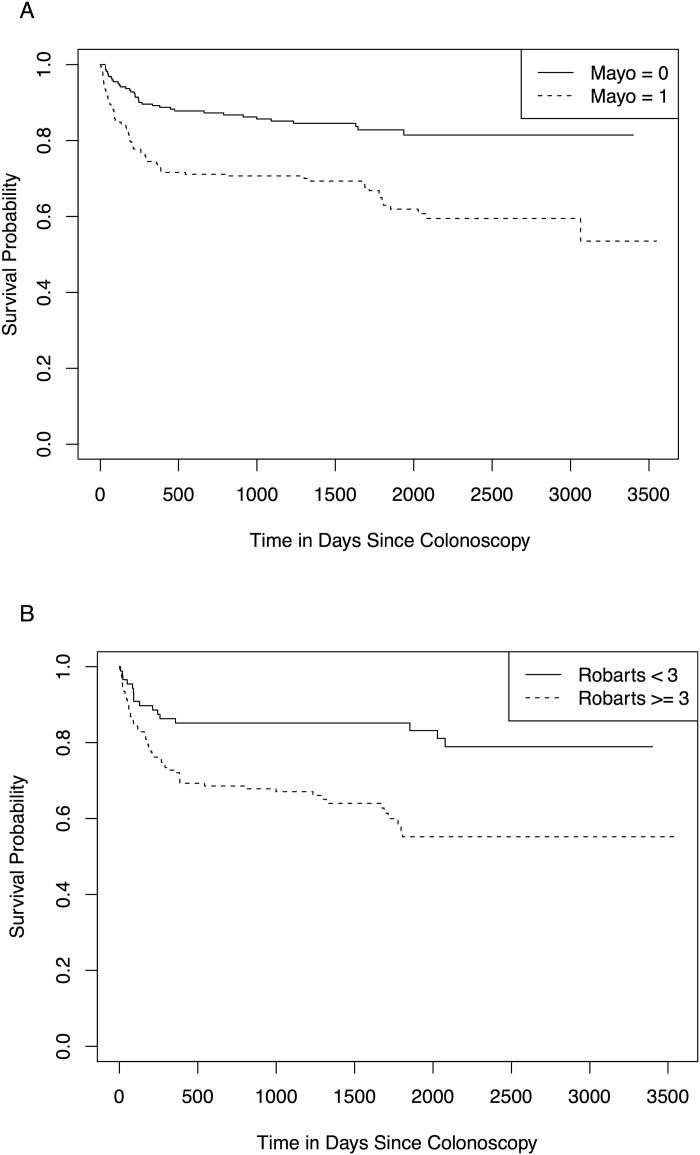
Survival curves of relapse rates for ulcerative colitis patients at the University of Maryland School of Medicine and New York University Langone Health based on stratification by Mayo Endoscopic Score (A) and Robarts Histopathologic Index (B) independently.

Clinical outcomes were assessed in extended clinical follow-up with a mean of 1364 days since the index colonoscopy to capture additional patients reaching the composite outcome. A total of 194 patients met the composite clinical outcome in extended follow-up. Eighty-three of these had an MES of 0 at the index colonoscopy, and the remaining 111 had an MES of 1. Using Cox proportional hazard ratios, an RHI greater than 3 was significantly associated with an increased risk of relapse in all patients, regardless of MES (HR = 1.88, 95% CI [1.41, 2.49], Figure 3A). Additionally, MES, regardless of RHI, was significant (HR = 2.43, 95% CI [1.63, 3.61], Figure 3B). Together, these results independently indicate that an MES of 1 or an RHI greater than 3 are predictors of subsequent relapse. Patients were stratified into 4 groups (MES 0, RHI ≤ 3; MES 0, RHI > 3; MES 1, RHI ≤ 3; MES 1, RHI > 3) and a logistic regression was performed controlling for age and sex. An RHI greater than 3 was predictive of relapse (*P*-value 0.008, Table 3). An MES of 1 alone was not predictive of relapse (*P*-value 0.126), however, the interaction between MES and RHI was significant, indicating RHI is more predictive of relapse in patients with an MES of 0 than an MES of 1 (p = 0.011) (Table 3). Time in the study was controlled for and had a small but significant impact on rate of relapse (*P*-value < 0.001), suggesting most relapses occur early in follow-up.

**Table 3.** Logistic regression model details of factors predicting relapse in patients with ulcerative colitis at the University of Maryland School of Medicine and New York University Langone Health.

Factor	Odd’s ratio	Confidence interval	*P*-value for the regression
Mayo score (MES 1 relative to MES 0)	2.234	(0.792,6.262)	0.126
Robart’s Histologic Index (RHI > 3 relative to RHI ≤ 3)	18.520	(2.264,163.586)	0.00805
Age	1.003	(0.981,1.026)	0.783
Sex (F relative to M)	1.123	(0.582,2.160)	0.727
Time in study	0.996	(0.995,0.997)	< 2^−16^
Interaction between MES and RHI Scores	0.043	(0.004,0.470)	0.0112

## Discussion

In this dual-center, retrospective study, UC patients with an MES of 0 (endoscopic remission) or 1 (endoscopic improvement) in histologic remission defined by an RHI less than or equal to 3 were less likely to experience subsequent clinical relapse. The presence of histologic inflammation in all patients and within each MES group was associated with an increased risk of relapse, including change in medical therapy, corticosteroid prescription, UC-related hospitalization, or colectomy. This trended to significance within 1-year post-index colonoscopy and reached significance when outcome data was extended to all follow-ups. Our data demonstrates conflicting results with a recent retrospective study by Narula et al.,^12^ which did not find a significant difference in time to relapse in patients classified in endoscopic remission that were further stratified to groups based on the presence of histologic activity. Both studies were completed at 2 tertiary academic IBD centers; however, our study utilized a validated scoring system with the RHI to define histologic remission, which may have represented a more stringent and consistent definition of histologic remission.

Other studies have supported our conclusion. A prior study by Lobaton et al. prospectively enrolled UC patients undergoing surveillance colonoscopy who were found to have an MES of 0 or 1 into a 12 month follow-up period. This study found that patients with active histologic disease as defined by the Geboes score had an increased risk of clinical relapse in 1 year,^13^ which correlates with our data; however, we used a different index to assess histologic activity (RHI) in our study. With prospectively collected data from 13 IBD centers in Portugal, Magro et al.^14^ demonstrated a statistically significant increase in clinical relapse and shorter time to relapse if histologic inflammation as defined by the Geboes score was present. Although these results are in support of our conclusions, this study differed from ours by including all asymptomatic patients in their analysis, including those with an MES greater than 1 at baseline. A large meta-analysis of 28 studies assessing risk of relapse in patients with histologic activity on colonic biopsies showed stronger associations between histologic activity and increased risk of relapse if a validated histologic scoring system was used.^15^ Interestingly, there was a larger impact of histologic activity on risk of relapse if studies included at least 24 months of follow-up post-colonoscopy, which supports our findings of significance when extending our outcome data beyond 1 year.

There are many indices for scoring histological inflammation, with a 2017 systematic review study of 126 studies finding that the Nancy Index and RHI have undergone the most validation.^8^ The RHI, specifically, employs a combination of chronic inflammatory infiltrate, neutrophil invasion of the lamina propria and epithelium, and the presence of ulcerations or erosions to develop an overall score of inflammation.^9^ When compared against other indices including the Geboes score and modified Riley score in a 2017 study of 48 UC colon biopsies, the RHI was found to have favorable operating properties as an alternative instrument for scoring colonic histologic inflammation.^9^

Our findings have important implications for the care provided to UC patients. Current standards of practice define remission based on endoscopic appearance as scored by the MES. Endoscopic remission or improvement is achieved when the score is less than or equal to one. However, there have been prior studies that have shown as many as 40% of UC patients in endoscopic remission will have histologic inflammation of varying degrees on biopsies.^16,17^ There have been multiple scoring systems validated in research populations to objectively define histologic remission,^8^ and histologic remission has been used in recent investigational trials as a secondary outcome of treatment success.^18,19^ Despite the expanded use in the research forum, histologic scoring has not been widely adopted in clinical practice. Our study provides further support for the value that is added by providing a validated histologic score and incorporating routine colonic biopsies for histologic staging in UC patients. Patients who are identified as having ongoing histologic inflammation despite reaching endoscopic remission or improvement may require enhanced clinical monitoring and/or medication intensification. enhanced clinical monitoring or treatment escalation to achieve histologic remission is associated with improved clinical outcomes. A barrier to this approach is that standard histologic scores are not widely available in clinical practice. We have an ongoing study to determine if a qualitative assessment of active inflammation by the gastroenterologist’s review of the pathology report is equally effective in stratifying patients as low and high risk for relapse.

Our study has strengths and weaknesses. To the best of our knowledge, our study is one of the largest studies to evaluate the risk of relapse based on histologic inflammation. A recently published large retrospective study by Seong et al.^20^ also demonstrated an increased risk of clinical relapse in patients with histologic inflammation as defined by an elevated Geboes score, supporting our results. Additionally, data were obtained from 2 large tertiary care IBD centers with expertise in both IBD and gastrointestinal pathology. All biopsies were reviewed by expert GI pathologists who provided a validated, objective score based on the RHI. This lends reproducibility and decreases the risk of inter-observer variability. Our study does have some limitations, most notably that it was a retrospective study. Due to the retrospective nature of data collection, there was a significant amount of missing data values when attempting to collect corresponding clinical data such as c reactive protein or sedimentation rate, or the presence of clinical remission based on validated scoring systems at time of colonoscopy. As both centers were referral centers, it is also likely that the patient populations are not representative of the community at large with UC. Additionally, we acknowledge the lack of centra reading for both endoscopy images and histology slides.

In conclusion, our study demonstrates that patients with an MES of 0 or 1 in histologic remission as defined by the RHI were less likely to experience subsequent clinical relapse. Our study highlights a signal that potentially not all patients in endoscopic remission/improvement as defined by the MES are equal, and that further enhanced monitoring strategies with routine disease activity assessment through biopsies may further risk stratify which patients are at increased risk for an adverse clinical outcome. Future studies are needed to verify our results, to determine if gastroenterologist’s interpretation of pathology reports is predictive of relapse, and if escalating medical therapy decreases relapse in patients with ongoing histologic activity.

## Supplementary Material

otad059_suppl_Supplementary_Figures_1

otad059_suppl_Supplementary_Tables_1-2

## Data Availability

The data underlying this article will be shared on reasonable request to the corresponding author.
