# Supplementary figures and images for: Histologic Inflammation can Predict Future Clinical Relapse in Ulcerative Colitis Patients in Endoscopic Remission
Source: Crohns Colitis 360. 2023 Oct 18;5(4):otad059. doi: 10.1093/crocol/otad059 (PMC10599395; doi:10.1093/crocol/otad059)

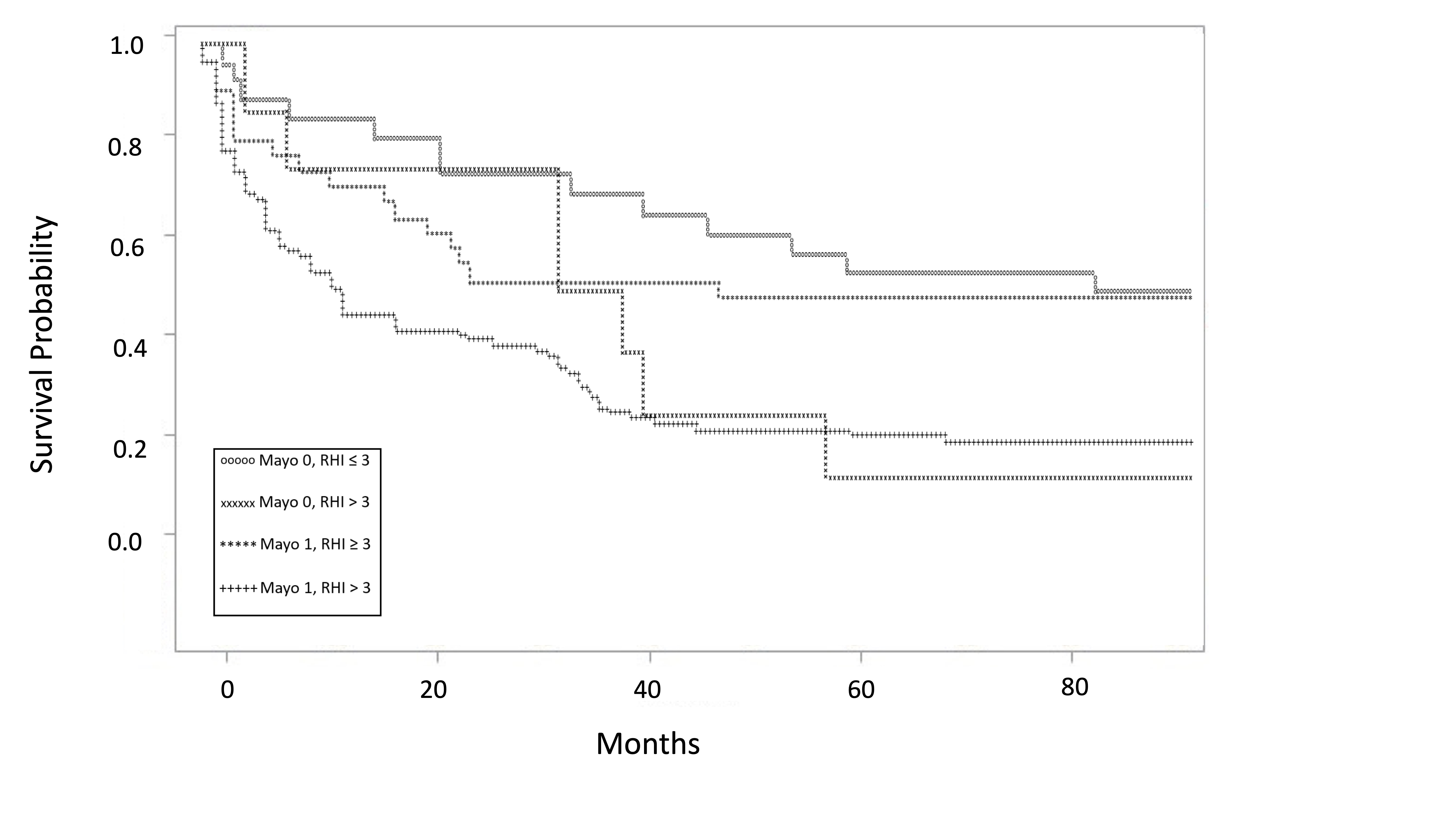

Supplement: otad059_suppl_Supplementary_Figures_1 [file otad059_suppl_supplementary_figures_1.jpeg]
