# Supplementary material for: Histologic Inflammation can Predict Future Clinical Relapse in Ulcerative Colitis Patients in Endoscopic Remission
Source: Crohns Colitis 360. 2023 Oct 18;5(4):otad059. doi: 10.1093/crocol/otad059 (PMC10599395; doi:10.1093/crocol/otad059)
Supplement: otad059_suppl_Supplementary_Tables_1-2 [file otad059_suppl_supplementary_tables_1-2.docx]

**SUPPLEMENTARY DATA:**

SUPPLEMENTARY TABLE 1:

| Variable  (n, %) | Mayo 0  (n=228) | Mayo 1  (n=218) | p-value |
| --- | --- | --- | --- |
| **Age**  ≤ 40  40-60  ≥ 60 | 135 (60)  60 (26)  32 (14) | 123 (57)  64 (29)  31 (14) | 0.64 |
| **Race**  Caucasian  African American  Other | 188 (83)  12 (5)  27 (12) | 158 (72)  26 (12)  34 (16) | 0.04 |
| **Sex**  Male  Female | 106 (47)  121 (53) | 106 (49)  112 (51) | 0.70 |
| **UC Phenotype**  Proctitis  Left-sided  Pan-colitis | 32 (15)  78 (35)  110 (50) | 55 (25)  73 (34)  90 (41) | <0.01 |
| **Smoking Status**  Never smoker  Former smoker  Current smoker | 155 (68)  57 (25)  15 (7) | 155 (70)  56 (27)  7 (3) | 0.25 |
| **Medications**  None  5-ASA  Immunomodulator  Anti-TNF  Vedolizumab  Other/Combination | 24 (11)  67 (30)  17 (7)  35 (15)  8 (4)  76 (33) | 18 (8)  96 (44)  18 (8)  33 (15)  10 (5)  43 (20) | <0.01 |
| **Relapse of Symptoms**  No Relapse  Relapse | 195 (86)  32 (14) | 153 (70)  65 (30) | <0.01 |
| **Steroid Course**  No Steroids  Steroids | 206 (91)  21 (9) | 173 (79)  45 (21) | <0.01 |
| **Hospitalization**  No Hospitalization  Hospitalization | 225 (99)  2 (1) | 212 (97)  6 (3) | 0.17 |
| **Colectomy**  No Colectomy  Colectomy | 227 (100)  0 (0) | 214 (98)  4 (2) | 0.05 |
| **ESR**  ≤ 30  >30 | 18 (86)  3 (14) | 25 (86)  4 (14) | 1.0 |
| **CRP**  ≤ 3  >3 | 110 (87)  16 (13) | 64 (78)  18 (22) | 0.08 |
| **UC duration (years)**  ≤ 10  >10 | 121 (57)  90 (43) | 133 (65)  73 (35) | 0.13 |

**Supplemental Table 1.** Demographic and clinical characteristics of patients with UC at the University of Maryland and New York University at time of baseline colonoscopy with endoscopic Mayo 0 score (n=228) or Mayo 1 score (n=218)

SUPPLEMENTARY TABLE 2:


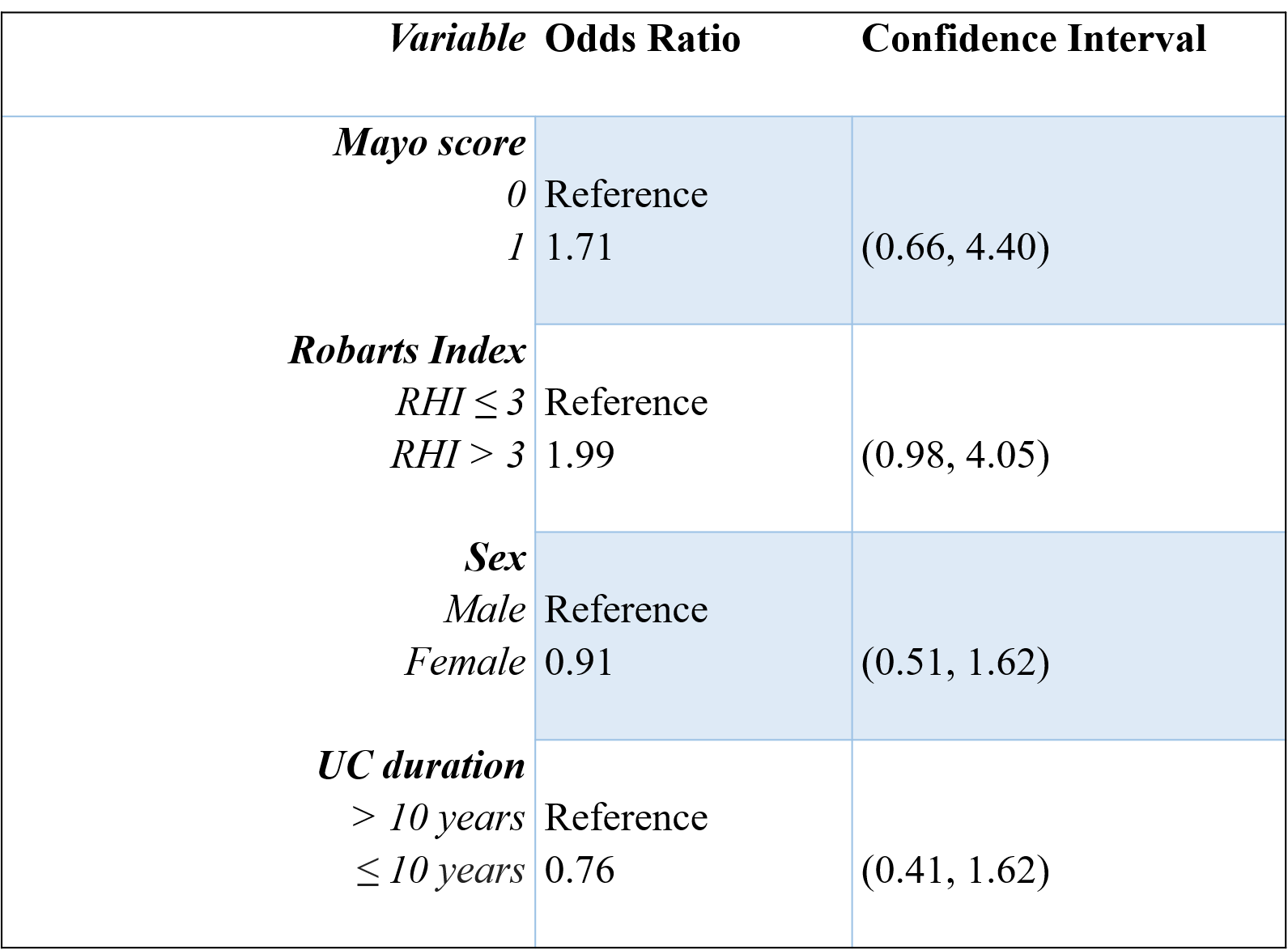


**Supplementary Table 2**. Odds of clinical relapse based on demographic and clinical variables for patients with ulcerative colitis at the University of Maryland School of Medicine and New York University Langone Health
